# Effects of AC induced electric fields on neuronal firing sensitivity and activity patterns

**DOI:** 10.3389/fncom.2025.1612314

**Published:** 2025-09-18

**Authors:** Chunhua Yuan, Rupei Chen, Xiangyu Li, Yueyang Zhao

**Affiliations:** ^1^School of Automation and Electrical Engineering, Shenyang Ligong University, Shenyang, China; ^2^Shengjing Hospital of China Medical University, Shenyang, China

**Keywords:** firing sensitivity, mean firing rate, AC induced electric field, bifurcation, two-compartment neuron

## Abstract

**Introduction:**

Understanding how neurons respond to time-varying electric fields is essential for both basic neuroscience and the development of neuromodulation strategies. However, the mechanisms by which alternating-current induced electric fields (AC-IEF) influence neuronal sensitivity and firing remain unclear.

**Methods:**

We developed a modified two-compartment Pinsky–Rinzel (PR) neuron model incorporating AC-IEF stimulation. Using systematic simulations, we examined firing responses across a wide range of field frequencies, amplitudes, and intrinsic membrane parameters, including inter-compartmental conductance and potassium reversal potential.

**Results:**

Neurons exhibited no firing or sensitivity when the field amplitude was less than twice the baseline membrane potential, regardless of conductance or reversal potential. Sensitivity increased markedly with amplitude: for example, when the amplitude exceeded 0.5 mV/cm, maximum firing rates rose by up to 45% and the sensitivity frequency range extended to 10–50 Hz. Phase-locking phenomena (1:1 and 2:1) were observed, with bandwidths widening as amplitude increased. For amplitudes below 30 mV, firing pattern transitions depended strongly on inter-compartmental conductance, whereas amplitudes ≥30 mV produced a consistent progression ending in subthreshold oscillations. Similar parameter-dependent transitions occurred for different potassium reversal potentials, converging at high amplitudes.

**Discussion:**

These results reveal a parameter-dependent mechanism by which AC-IEF modulate neuronal excitability. The findings provide qualitative rather than strictly quantitative insights into how external electromagnetic environments can shape neural activity, offering new directions for targeted neuromodulation in both health and disease.

## 1 Introduction

Theoretical and practical significance of external electric fields is becoming increasingly prominent in neural modulation studies. They exert significant influence on neuronal firing and network dynamics by regulating membrane potential and ion channel kinetics (Yan et al., 2020). AC-IEF can modulate the phase of local field potentials and show high sensitivity to neuronal structural coupling and directional organization (Asan et al., 2020; Wischnewski et al., 2024). Under electromagnetic modulation, critical mechanisms including calcium channels display intricate dynamics (Bertagna et al., 2021). Notably, neurons and electric fields are strongly bidirectionally coupled (Zandi-Mehran et al., 2020). However, the mechanisms underlying these interactions remain poorly understood in multi-compartmental models and complex neural networks (Kafraj et al., 2020). A deeper investigation of neuronal sensitivity to AC-IEF is essential for elucidating response patterns to electric fields and improving techniques like transcranial alternating current stimulation.

The sensitivity of neurons to external stimuli is governed by electrophysiological properties—including ion channel dynamics, membrane capacitance, and threshold potential—and is further modulated by local network architecture and environmental state (Shen et al., 2024). When exposed to AC-IEF, neurons display nonlinear response behaviors such as altered phase responsiveness, modulation of firing frequency, and firing pattern reorganization, demonstrating their strong adaptability to subtle external perturbations (Kafraj et al., 2020). Different neural systems show diverse sensitivity profiles: cortical neurons adjust input gain through excitatory-inhibitory pathways and state-dependent variables, whereas mechanosensory neurons achieve efficient signal conversion through specialized structures and highly responsive ion channels (Handler and Ginty, 2021). A systematic investigation into neuronal sensitivity across diverse physiological states is crucial for elucidating regulatory mechanisms and offering theoretical foundations for AC-IEF and neuromodulatory interventions such as transcranial alternating current stimulation (tACS) (Du et al., 2023; Cao et al., 2024).

The two-compartment neuron model partitions the neuron into somatic and dendritic domains, enabling precise characterization of subregional coupling dynamics. The Pinsky–Rinzel (PR) model, built upon robust biophysical principles and exhibiting rich dynamical behavior, is extensively employed to simulate a wide range of electrical activities including fast bursting, single spike firing, and resting states (Rezvani-Ardakani et al., 2020). When subjected to AC-IEF, the model demonstrates pronounced sensitivity to spatial gradients and directional variations, revealing complex somato-dendritic responses under heterogeneous stimulation. Owing to its structural strengths, it serves as an ideal framework for investigating neuronal sensitivity to electric field modulation and transitions in oscillatory modes (Xia et al., 2021). With the integration of multi-scale approaches in neuroscience—including neurotransmitter modeling, brain tissue engineering, and genetic interventions—the PR model serves as a critical link between cellular mechanisms and system-level functions in EEG modulation, epilepsy intervention, and optimization of electrical stimulation protocols (Mokhtare et al., 2022; Alfihed et al., 2024). Recent EEG-based research has demonstrated practical applications in automated seizure detection via mobile platforms (Lasefr et al., 2023) and in classifying visually evoked emotions through advanced frequency-domain analysis (Adhikari et al., 2025).

Most existing studies have concentrated on the influence of specific chemical or physical factors on neuronal sensitivity, including chemical composition and the angle or direction of external stimulation. Although neuronal sensitivity under electrical stimulation has been explored to some extent, investigations targeting neuronal firing sensitivity in response to AC-IEF remain relatively scarce.

Building upon this background, we hypothesize that the PR neuron is highly sensitive to AC-IEF, and that its firing behavior and sensitivity are significantly modulated by two key parameters: the somato-dendritic coupling conductance (*g*_c_) and the potassium reversal potential (*V*_k_). To test this hypothesis, we developed a two-compartment PR model subjected to AC-IEF, using the average firing rate (*F*) as a metric of neuronal output. We then systematically analyzed the effects of varying electric field amplitudes and frequencies on the influence of *g*_c_ and *V*_k_ on firing dynamics and bifurcation behavior. A three-dimensional firing-amplitude-frequency (*F*-*A*-*F*_req_) surface was constructed, followed by *F*-*F*_req_ dependency curves and two-dimensional *F*-*g_c_*-*F*_req_ and *F*-*V*_k_*-F*_req_ mappings under different amplitude conditions. These visualizations reveal how model parameters sculpt the response patterns and sensitivity boundaries of the neuron to AC-IEF.

Although this study focuses on a biophysically realistic PR neuron model and does not encompass the full diversity of neuronal morphologies or *in vivo* network effects, it offers several notable contributions: (1) Systematic sensitivity analysis—it investigates the firing sensitivity of PR neurons under AC-IEF, revealing nonlinear responses to field stimuli across different frequencies and amplitudes. (2) Parameter-based modulation—it incorporates somato-dendritic coupling conductance (*g*_c_) and potassium reversal potential (*V*_k_) as key regulatory parameters, clarifying the respective roles of structural coupling and ionic dynamics in shaping neuronal responses to electric fields. (3) Comprehensive multi-dimensional mapping—it develops mappings centered on the average firing rate (*F*)—including *F*-*A*-*F*_req_, *F-F*_req_, *F*-*g*_c_-*F*_req_, and *F*-*V*_k_-*F*_req_—offering theoretical perspectives on how complex neuronal architectures dynamically adapt to non-uniform electric fields. The remainder of this paper is organized as follows: Section 2 describes the PR neuron model and the implementation of AC-IEF stimulation; Section 3 presents the simulation results and sensitivity analyses; Section 4 discusses the implications and limitations; and Section 5 concludes the study.

## 2 Methods

### 2.1 The PR model

**Figure 1:**
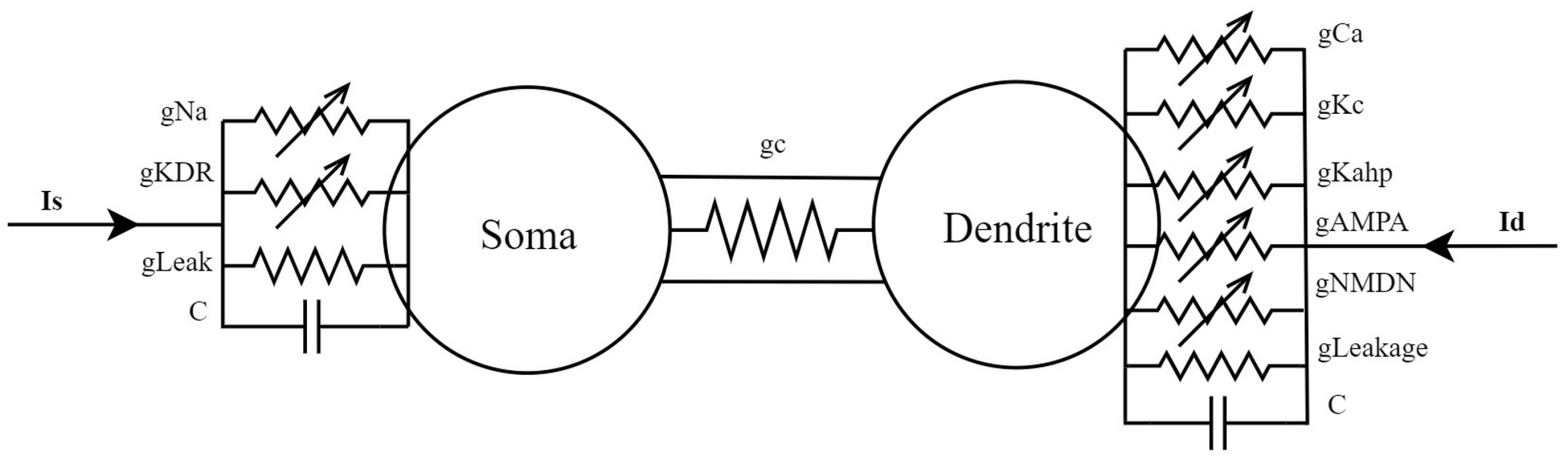
A simplified cable model for a two-compartment neuron located in the hippocampal CA3 region.

The PR model’s dynamical properties (Pinsky and Rinzel, 1995) are determined by a set of coupled nonlinear differential equations, encompassing a current balance equation that captures variations in membrane potential, along with activation and inactivation variables for ion channel dynamics. The simplified cable model of this system is illustrated in Figure 1. The soma contains fast sodium channels and delayed rectifier potassium channels for generating action potentials, whereas the dendrite features slow calcium channels and calcium-dependent potassium channels to modulate the rhythmicity of burst firing. The soma and dendrite are connected via conductance coupling, permitting current flow in both directions and facilitating bidirectional signal transmission. The model additionally incorporates dynamic variations in calcium concentration to describe the slower time-scale processes in the dendritic region. Two major parameters influencing the system are the coupling conductance (*g*_c_) and the potassium channel reversal potential (*V*_k_), both adjustable and open to further exploration in the model.

The PR model consists of the following differential equations (see Equations 1 and 2):


(1) $$\begin{array}{l} C_{m}{\dot{V}}_{s}=-I_{\text{sLeak}}(V_{s})-I_{\mathrm{Na}}(V_{s},h)-I_{\mathrm{KDR}}(V_{s},n)\\ +\frac{g_{c}(V_{d}-V_{s})}{\rho }+\frac{I_{s}}{\rho } \end{array}$$

(2) $$\begin{array}{l} C_{m}{\dot{V}}_{d}=-I_{\text{dLeak}}(V_{d})-I_{\mathrm{Ca}}(V_{d},s)-I_{\text{KAHP}}(V_{d},q)\\ -I_{\mathrm{KC}}(V_{d},\mathrm{Ca},c)+\frac{g_{c}(V_{s}-V_{d})}{1-\rho }+\frac{I_{d}}{1-\rho } \end{array}$$

where: *C*_m_ denotes the membrane capacitance, with a value of 3.0 μF/cm^2^; *V*_s_ and *V*_d_ represent the somatic and dendritic membrane potentials (mV), respectively; *I*_sLeak_ is the somatic leak current; *I*_Na_ is the fast sodium current in the soma; *I*_KDR_ is the delayed rectifier potassium current in the soma; *g*_c_ is the somato-dendritic coupling conductance (mS/cm^2^); *ρ* denotes the proportion of the somatic membrane area to the total membrane area, with a value of 0.5; *I*_s_ is the externally applied current to the soma (μA/cm^2^); *I*_dLeak_ is the dendritic leak current; *I*_Ca_ is the high-threshold calcium current in the dendrite; *I*_KAHP_ is the calcium-dependent slow afterhyperpolarization potassium current in the dendrite; and *I*_KC_ is the calcium-activated fast potassium current in the dendrite.

The expressions for the ionic currents are:


$$I_{\text{sLeak}}=g_{L}(V_{S}-V_{L}),$$

$$I_{\mathrm{Na}}=g_{\mathrm{Na}}m_{\infty }^{2}h(V_{S}-V_{\mathrm{Na}}),$$

$$I_{\mathrm{KDR}}=g_{\mathrm{KDR}}n(V_{s}-V_{k}),$$

$$\;I_{\mathrm{DS}}^{\text{in}}=g_{C}(V_{d}-V_{S}),$$

$$\;I_{\text{dLeak}}=g_{L}(V_{d}-V_{L}),$$

$$\;I_{\mathrm{Ca}}=g_{\mathrm{Ca}}s^{2}(V_{d}-V_{\mathrm{Ca}}),$$

χ(Ca)=min(Ca/250,1),

$$I_{\mathrm{KC}}=g_{\mathrm{KC}}\mathrm{c\chi}(\mathrm{Ca})(V_{d}-V_{k}),$$

$$\;I_{\text{KAHP}}=g_{\text{KAHP}}q(V_{d}-V_{K}).$$

In the above equation block, *g*_L_ denotes the membrane leak conductance, with a value of 0.1 mS/cm^2^; *g*_Na_ represents the maximal sodium conductance in the soma, with a value of 30 mS/cm^2^; *g*_KDR_ denotes the maximal delayed rectifier potassium conductance in the soma, with a value of 15 mS/cm^2^; *g*_Ca_ is the maximal high-threshold calcium conductance in the dendrite, with a value of 10 mS/cm^2^; *g*_KAHP_ represents the maximal calcium-dependent slow afterhyperpolarization potassium conductance in the dendrite, with a value of 0.8 mS/cm^2^; *g*_KC_ denotes the maximal calcium-activated fast potassium conductance in the dendrite, with a value of 15 mS/cm^2^; *g*_NMDA_ represents the maximal synaptic conductance mediated by NMDA-type glutamate receptors, with a value of 0.03 mS/cm^2^; and *g*_AMPA_ denotes the maximal synaptic conductance mediated by AMPA-type glutamate receptors, with a value of 0.0045 mS/cm^2^.

The dynamics of the gating variables for each ion channel are governed by (see Equation 3):


(3) $$\frac{dx}{dt}=\frac{x_{\infty }(V)-x}{\tau_{x}(V)}.$$

Specifically, $x_{\infty }=\frac{\alpha_{x}}{\left(\alpha_{x}+\beta_{x}\right)}$ and $\tau_{x}=\frac{1}{\left(\alpha_{x}+\beta_{x}\right)}$, where (*x* = m, h, n, s, c, q), m represents the activation gate of the sodium (Na) channel, controlling the probability of channel opening and thus the rise of the action potential. The h represents the inactivation gate of the sodium channel, regulating channel closing and determining the termination of sodium influx after the peak of the action potential. The n corresponds to the activation gate of the delayed rectifier potassium (K_DR) channel, which regulates K^+^ currents and participates in action potential repolarization and hyperpolarization. The s represents the activation gate of the calcium-dependent potassium (K_Ca) channel, whose dynamics are regulated by the intracellular calcium concentration, influencing slow afterhyperpolarization and pulse rate adaptation. The c represents the intracellular calcium concentration in the dendritic region and is a key variable for calcium-dependent currents. The q represents the activation gate of the low-threshold calcium (CaT) channel, controlling dendritic calcium influx and playing a key role in burst firing and biphasic oscillatory behavior. The dynamic characteristics of each gating variable (m, h, n, s, q) are determined by the corresponding rate functions *α* and *β*, where *α* represents the opening rate and *β* represents the closing rate (see Table 1 for details).

**Table 1:** Rate functions of the gating variables for each ion channel.

Variable	Forward (*α*)	Backward (*β*)
m	$\alpha_{m}=\frac{0.32(13.1-V_{s})}{\exp((13.1-V_{s})/4)-1}$	$\beta_{m}=\frac{0.28(V_{s}-40.1)}{\exp(V_{s}-40.1)/5)-1}$
h	$\alpha_{h}=0.128\exp(\frac{17.0-V_{s}}{18.0})$	$\beta_{h}=\frac{4}{1+\exp((40.0-V_{s})/5)}$
n	$\alpha_{n}=\frac{0.016(35.1-V_{s})}{\exp((35.1-V_{s})/5)-1}$	$\beta_{n}=0.25\exp{\left(0.5-0.025V_{s}\right)}^{n}$
S	$\alpha_{s}=\frac{1.6}{1+\exp(-0.072(V_{d}-65))}$	$\beta_{s}=\frac{0.02(V_{d}-51.1)}{\exp((V_{d}-51.1)/5)-1}$
C	$\alpha_{c}=\frac{\exp((V_{d}-10.0)/11-(V_{d}-6.5)/27)}{18.975}$ $\alpha_{c}=2\exp(\frac{6.5-V_{d}}{27})$	$\beta_{c}=2\exp(\frac{6.5-V_{d}}{27})-\alpha_{c}$ $V_{d}\leq 50.0$ $\beta_{c}=0\;V_{d}>50.0$
q	$\alpha_{q}=\min(0.00002\mathrm{Ca},0.01)$	$\beta_{q}=0.001$

The dynamic equation for calcium ion concentration is as follows (see Equation 4):


(4) $$\mathrm{dCa}/dt=-0.13I_{\mathrm{Ca}}-0.075\mathrm{Ca},$$

where dCa/d*t* denotes the rate of change of calcium concentration (μM/ms); *I*_Ca_ is the high-threshold calcium current in the dendrite (μA/cm^2^). The values of the remaining variable parameters are presented in Table 2.

**Table 2:** Parameter values for the Pinsky–Rinzel model.

Parameters	Values
The membrane area of the soma region, ρ (proportional area)	0.5
Total membrane area, Area	6 × 10^−6^ (${\mathrm{cm}}^{2}$) (somatic radius: 5 μm)
Coupling conductance between the two compartments, $g_{c}$	1, 2.1, 5, 10, 15, 25 ($\mathrm{mS}/{\mathrm{cm}}^{2}$)
Membrane capacitance, $C_{m}$	3.0 $\mathrm{\mu F}/{\mathrm{cm}}^{2}$
Reversal potential of sodium ions, $V_{\mathrm{Na}}$	120 (mV)
Reversal potential of calcium ions, $V_{\mathrm{Ca}}$	140 (mV)
Reversal potential of potassium ions, $V_{K}$	−15 (mV)
Leak current potential, $V_{L}$	0 (mV)
Synaptic potential, $V_{\mathrm{syn}}$	60 (mV)
Leak conductance, $g_{L}$	0.1 ($\mathrm{mS}/{\mathrm{cm}}^{2}$)
Sodium conductance, $g_{\mathrm{Na}}$	30 ($\mathrm{mS}/{\mathrm{cm}}^{2}$)
Delayed rectifier potassium conductance, $g_{\mathrm{KDR}}$	15 ($\mathrm{mS}/{\mathrm{cm}}^{2}$)
Calcium conductance, $g_{\mathrm{Ca}}$	2.1 ($\mathrm{mS}/{\mathrm{cm}}^{2}$)
Potassium afterhyperpolarization conductance, $g_{\text{KAHP}}$	0.8 ($\mathrm{mS}/{\mathrm{cm}}^{2}$)
Potassium conductance, $g_{\mathrm{KC}}$	15 ($\mathrm{mS}/{\mathrm{cm}}^{2}$)
Somatic current, $I_{s}$	0 ($\mathrm{\mu A}/{\mathrm{cm}}^{2}$)
Dendritic current, $I_{d}$	0.7 ($\mathrm{\mu A}/{\mathrm{cm}}^{2}$)

### 2.2 Establishment of the PR neuron model under the influence of a AC-IEF

According to Wang et al. (2013) and Ma et al. (2018), when a sinusoidal induced electric field with amplitude A and frequency ω is applied to the neuron, the membrane depolarization voltage ΔV(t) satisfies (see Equation 5):


(5) ΔV(t)=λAsinωt

In this equation, λ represents the polarization length of the neuron. Considering the depolarization ΔV(t) of the cell membrane as an external perturbation applied to the membrane potential V(t) (Mukherjee et al., 2023), the PR model is then modified from Equations 1, 2 to Equations 6, 7:


(6) $$\begin{array}{l} C_{m}\frac{d(V_{s}+\mathrm{\Delta V})}{dt}=-I_{\text{sLeak}}(V_{s}+\Delta V)-I_{\mathrm{Na}}(V_{s}+\Delta V,h)\\ -I_{\mathrm{KDR}}(V_{s}+\Delta V,n)+\frac{g_{c}(V_{d}-V_{s})}{\rho }+\frac{I_{s}}{\rho }, \end{array}$$

(7) $$\begin{array}{l} C_{m}\frac{d(V_{d}+\mathrm{\Delta V})}{dt}=-I_{\text{dLeak}}(V_{d}+\mathrm{\Delta V})-I_{\mathrm{Ca}}(V_{d}+\mathrm{\Delta V},s)\\ -I_{\text{KAHP}}(V_{d}+\mathrm{\Delta V},q)-I_{\mathrm{kc}}(V_{d}+\mathrm{\Delta V},\mathrm{Ca},c)\\ +\frac{g_{c}(V_{s}-V_{d})}{1-\rho }+\frac{I_{d}}{1-\rho }. \end{array}$$

The induced electric field in this study is an alternating current (AC) induced electric field, with an electrode spacing d of 5 cm and the electric field strength E as follows (see Equation 8):


(8) $$E=\frac{V_{e}}{d}=\frac{A}{0.05\omega }\sin(\mathrm{\omega t})$$

Therefore, the voltage $V_{e}$ applied to the neuron is given by (see Equation 9):


(9) $$\;V_{e}=d\times E=\frac{A}{\omega }\sin(\mathrm{\omega t})$$

In the equation, ω represents the angular frequency, given by $2\pi F_{\mathrm{req}}$, where $F_{\mathrm{req}}$ is the frequency of the alternating current induced electric field, and A is the amplitude of the field. In this study, let $\Delta V=V_{e}$, and thus, the induced current variable $I_{e}$ under the alternating current induced electric field is(see Equation 10):


(10) $$I_{e}=C_{m}\frac{d(\mathrm{\Delta V})}{dt}=C_{m}A\cos(\mathrm{\omega t})$$

In summary, the improved PR neuron model incorporating the alternating current induced electric field is as follows (see Equations 11 and 12):


(11) $$\begin{array}{l} C_{m}{\dot{V}}_{s}=-I_{e}(A,\omega )-I_{\text{sLeak}}(V_{s}+V_{e})-I_{\mathrm{Na}}(V_{s}+V_{e},h)\\ -I_{\mathrm{KDR}}(V_{s}+V_{e},n)+\frac{g_{c}(V_{s}-V_{d})}{\rho }+\frac{I_{s}\;}{\rho }, \end{array}$$

(12) $$\begin{array}{l} C_{m}{\dot{V}}_{d}=-I_{e}(A,\omega )-I_{\text{dLeak}}(V_{d}+V_{e})-I_{\mathrm{Ca}}(V_{d}+V_{e},s)\\ -I_{\text{KAHP}}(V_{d}+V_{e},q)-I_{\mathrm{KC}}(V_{d}+V_{e},\mathrm{Ca},c)\\ +\frac{g_{c}(V_{s}-V_{d})}{1-\rho }+\frac{I_{d}}{1-\rho }. \end{array}$$

The model equations were numerically integrated on the MATLAB platform using a custom implementation of the classical fourth-order Runge–Kutta scheme. A fixed integration step of 0.1 ms was employed, with a total simulation duration of 7,000 ms. This choice ensured numerical stability and reproducibility, while avoiding the adaptive step-size adjustments inherent in MATLAB’s built-in solvers such as ode45. All other parameter values and variable expressions were consistent with the standard PR model.

### 2.3 Analysis of the firing activity of PR neurons under different influencing parameters

The neuron’s firing behavior under AC-IEF is shaped by both the soma-dendrite coupling conductance *g*_c_ and the potassium reversal potential *V*_k_. Variation in *g*_c_ modulates excitability and alters firing patterns, thereby influencing the neuron’s responsiveness to external fields. Meanwhile, changes in *V*_k_, regulated by extracellular potassium levels, shift the excitability threshold and affect sensitivity to electric field perturbations. This study investigates how these parameters govern the PR neuron’s firing dynamics and its sensitivity to AC-IEF.

**Figure 2:**
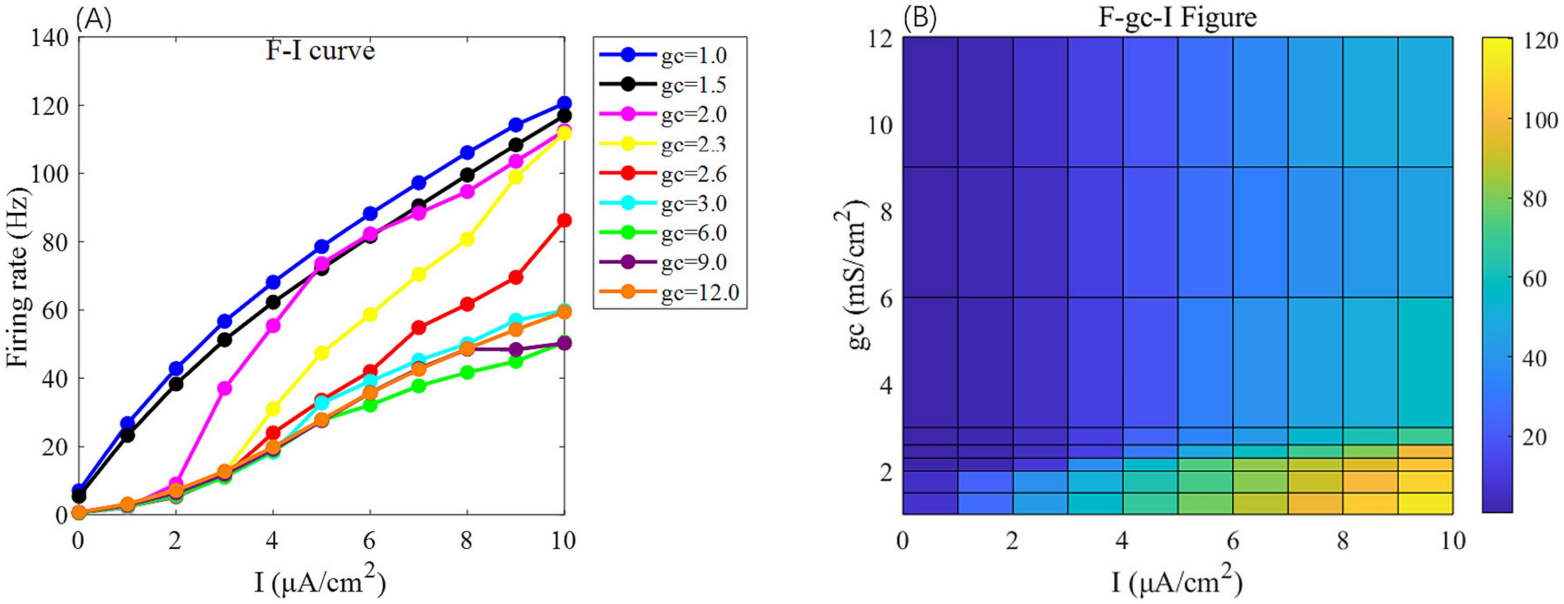
The *F-I* curve (left) and the *F-g*_c_*-I* plane diagram (right) of PR neurons under AC input. The model parameter is set to *V*_k_ = −15 mV. In the *F-I* curve, different colors correspond to neuronal firing frequencies under varying interconnection conductance (*g*_c_) values. The *g*_c_ value range is {1, 1.5, 2.0, 2.3, 2.6, 3.0, 6, 9, 12} mS/cm^2^. In the *F-g*_c_*-I* plane diagram, the horizontal axis represents the input current *I*, ranging from 0 to 10 μA/cm^2^ with a step size of 1 μA/cm^2^, while the vertical axis represents interconnection conductance *g*_c_, where different colors indicate distinct firing rates *F*.

**Figure 3:**
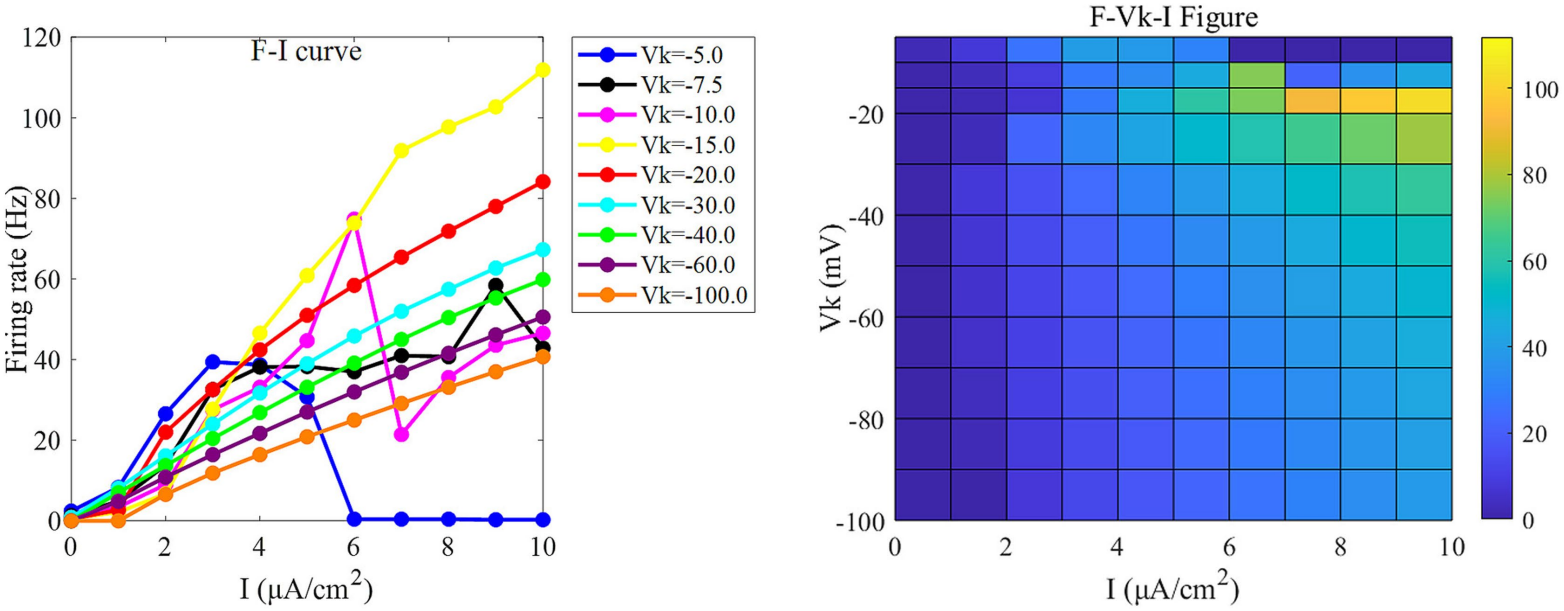
The *F-I* curve (left) and the *F-V*_k_*-I* plane diagram (right) of PR neurons under AC input. The model parameter is set to *g*_c_ = 2.1 mS/cm^2^. In the *F-I* curve, different colors correspond to neuronal firing frequencies under varying potassium channel reversal potentials (*V*_k_). *V*_k_ ranges from [−5, −7.5, −10, −15, −20, −30, −40, −60, −100] mV. In the *F*-*V*_k_-*I* plot, the horizontal axis represents the input current *I*, which ranges from 0 to 10 μA/cm^2^ in 1 μA/cm^2^ steps; the vertical axis represents the potassium channel reversal potential *V*_k_, which ranges from [−5, −10, −15, −20, −30, −40, −50, −60, −70, −80, −90, −100] mV. Different colors represent different firing rates *F*.

In the absence of DC stimulation $\left(I_{s}=0\;\mathrm{\mu A}/{\mathrm{cm}}^{2},I_{d}=0\;\mathrm{\mu A}/{\mathrm{cm}}^{2}\right)$, the neuron exhibits low firing frequency across parameter settings (as shown at *x* = 0 in Figures 2, 3), limiting the assessment of responses to external electric fields. To establish a consistent baseline, a weak DC stimulus $\left(I_{s}=0\;\mathrm{\mu A}/{\mathrm{cm}}^{2},I_{d}=0\;\mathrm{\mu A}/{\mathrm{cm}}^{2}\right)$ is applied.

**Figure 4:**
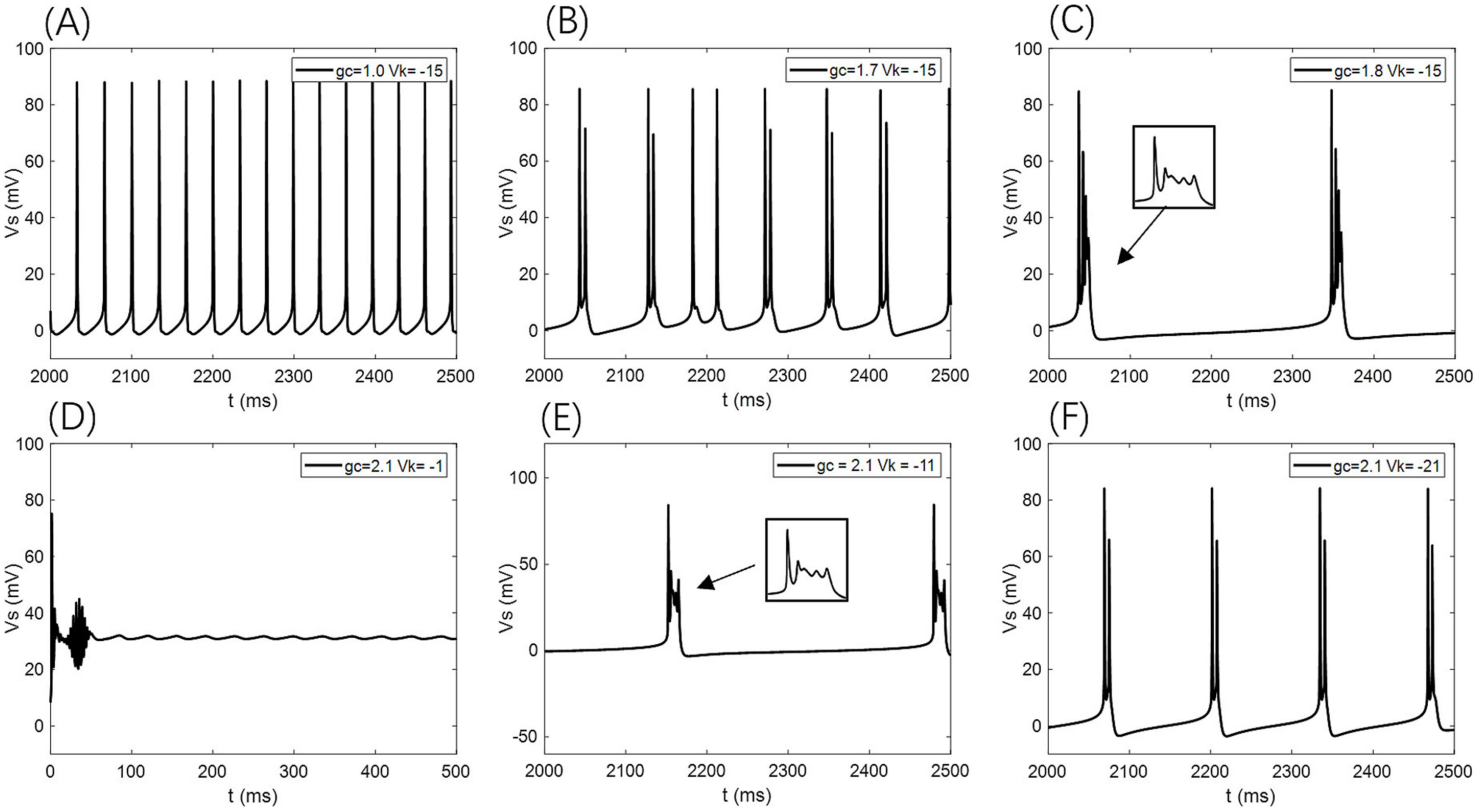
Different firing states of PR neurons: **(A)** periodic spike firing state, **(B)** burst-spike alternating firing state, **(C)** periodic burst firing state, **(D)** subdomain oscillation state, **(E)** periodic burst firing state, **(F)** double-period burst firing state.

Under this condition, the PR model exhibits diverse firing patterns depending on the coupling conductance *g*_c_ and potassium reversal potential *V*_k_. With *V*_k_ = −15 mV, firing behavior changes significantly across different *g*_c_ values: periodic spike firing occurs for 0 < *g*_c_ < 1.7 mS/cm^2^; cluster-spike alternation appears at *g*_c_ = 1.7 mS/cm^2^; periodic fast bursting emerges for 1.8 ≤ *g*_c_ < 10 mS/cm^2^; spike firing recurs for 10 ≤ *g*_c_ < 20 mS/cm^2^; and firing ceases when *g*_c_ ≥ 20 mS/cm^2^ (Figures 4A–C).

When *g*_c_ is set to 2.1 mS/cm^2^, the firing patterns also vary with *V*_k_: for *V*_k_ > −5 mV, the neuron exhibits subthreshold oscillations; within −22 mV < *V*_k_ ≤ −5 mV, it enters a periodic fast bursting state, with two-period bursting at *V*_k_ = −21 mV (Figures 4D–F); from −91 mV < *V*_k_ ≤ −22 mV, periodic spike firing dominates; and at *V*_k_ ≤ −91 mV, the neuron returns to rest.

To examine sensitivity to AC-IEF, representative values of *g*_c_ = 1, 1.7, 1.8 and 10 mS/cm^2^ are selected. Similarly, for *V*_k_, the values −5, −21 and −22 mV are chosen for analysis.

### 2.4 Explanation of the AC electric field

**Figure 5:**
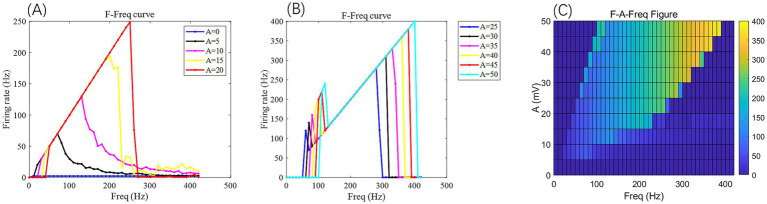
Under the AC-IEF, the firing frequency and electric field frequency curve *F-F*_req_ (curve) of PR neurons at different electric field voltage amplitudes (left) and the *F-A-F*_req_ two-dimensional mapping (right). The model parameters are *g*_c_ = 2.1 mS/cm^2^, *V*_k_ = −15 mV. In the *F-F*_req_ curve, different colored curves represent the firing frequency of neurons at different electric field frequencies for varying electric field amplitudes *A*. In the *F-A-F*_req_ plane diagram, the horizontal axis represents the electric field frequency F_req, ranging from 0 to 420 Hz, with a step size of 10 Hz, while the vertical axis represents the electric field amplitude *A*, where different colors represent distinct firing rates *F*.

Before examining the sensitivity of neurons to the AC-IEF, we begin by offering the following clarifications concerning the AC-IEF employed in this study:

(1) The AC-IEF is represented as $V_{e}=A\sin(\mathrm{\omega t})/\omega =A\sin(2\pi F_{\mathrm{req}}t)/(2\pi F_{\mathrm{req}})$, with A and $F_{\mathrm{req}}$ serving as adjustable parameters.
(2) The AC-IEF amplitude is defined as $A/(2\pi F_{\mathrm{req}})$, with *A* indicating its maximum possible amplitude, $A/(2\pi F_{\mathrm{req}})$ is the maximal amplitude since the sine function ranges between −1 and 1.
(3) Since the sine waveform of AC-IEF naturally fluctuates between positive and negative values, this study focuses exclusively on the positive amplitudes (*A* ≥ 0) of the AC field. This simplification allows us to isolate and analyze the excitatory effects of depolarizing field phases on the somatic membrane potential without the confounding influence of hyperpolarizing phases. Such an approach facilitates a clearer understanding of the mechanisms by which AC-IEF modulates neuronal firing rates and bifurcation dynamics under controlled stimulation conditions. However, it is important to acknowledge that real AC fields are bidirectional and comprise both depolarizing and hyperpolarizing components, which interact complexly with neuronal membranes and ion channels. Ignoring the negative half-cycles may overlook phenomena such as rebound excitation, phase locking, and more nuanced spike timing modulations that arise from the full oscillatory cycle. Therefore, the results presented here represent an idealized scenario resembling rectified or unidirectional stimulation patterns rather than fully physiological AC field waveforms. Future work should incorporate the complete bipolar AC waveform to capture the comprehensive neuronal response and better simulate *in vivo* conditions, thereby enhancing the translational relevance of computational models to neuromodulatory interventions such as transcranial alternating current stimulation (tACS).
(4) Most studies on isolated hippocampal slices have indicated that the safe range for the electric field intensity E is (0.1−1)V/m, within which the firing patterns of CA3 neurons can be regulated (Radman et al., 2009; Bikson et al., 2017; Grossman et al., 2019). Therefore, the maximum safe voltage applied to the neurons is $V_{\text{emax}}=1(V/m)\times 0.05\;m=0.05\;V=50\;\mathrm{mV}$, and the maximum value for the amplitude of the AC electric field, *A*, is within the range of [0,50]mV. As illustrated in Figure 5, when *A* is 50 mV, the neuronal firing frequency ceases after exceeding 800 Hz. Hence, in this study, the firing frequency of the AC electric field, $F_{\mathrm{req}}$, is within the range of [0,420]Hz.

## 3 Results

### 3.1 Effects of *g*_c_ on the firing sensitivity of neurons under a AC-IEF

**Figure 6:**
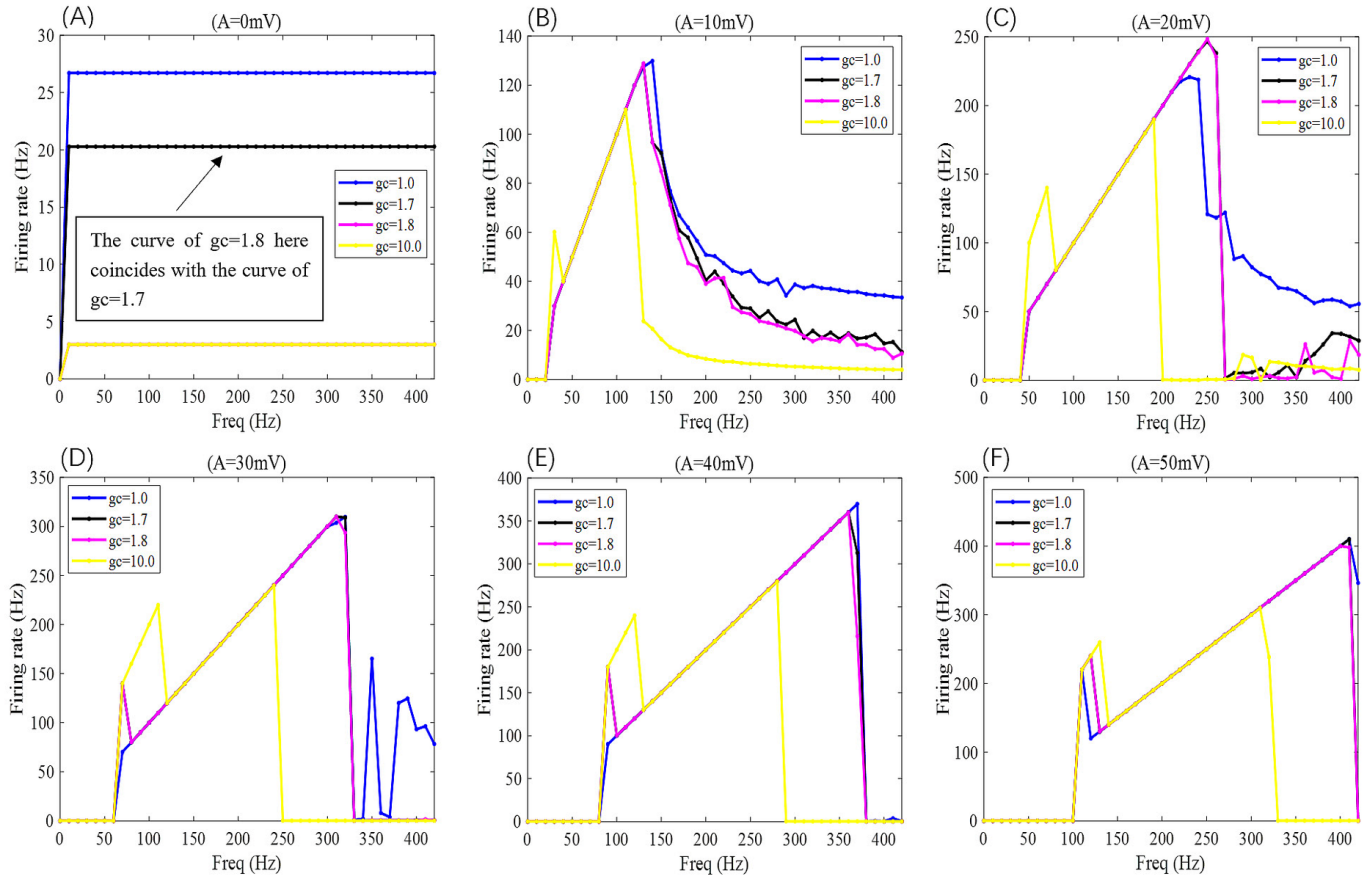
When subjected to weak DC stimulation, the *F*-*F*_req_ curves of PR neurons under the action of sinusoidal alternating electric fields with different coupling conductances *g*_c_ and varying electric field amplitudes A, where *g*_c_ = 1, 1.7, 1.8, and 10 mS/cm^2^, and the electric field amplitudes A take values of 0, 10, 20, 30, 40, and 50 mV, with the frequency of the AC-IEF ranging from 0 to 420 Hz, with a 10 Hz interval.

**Figure 7:**
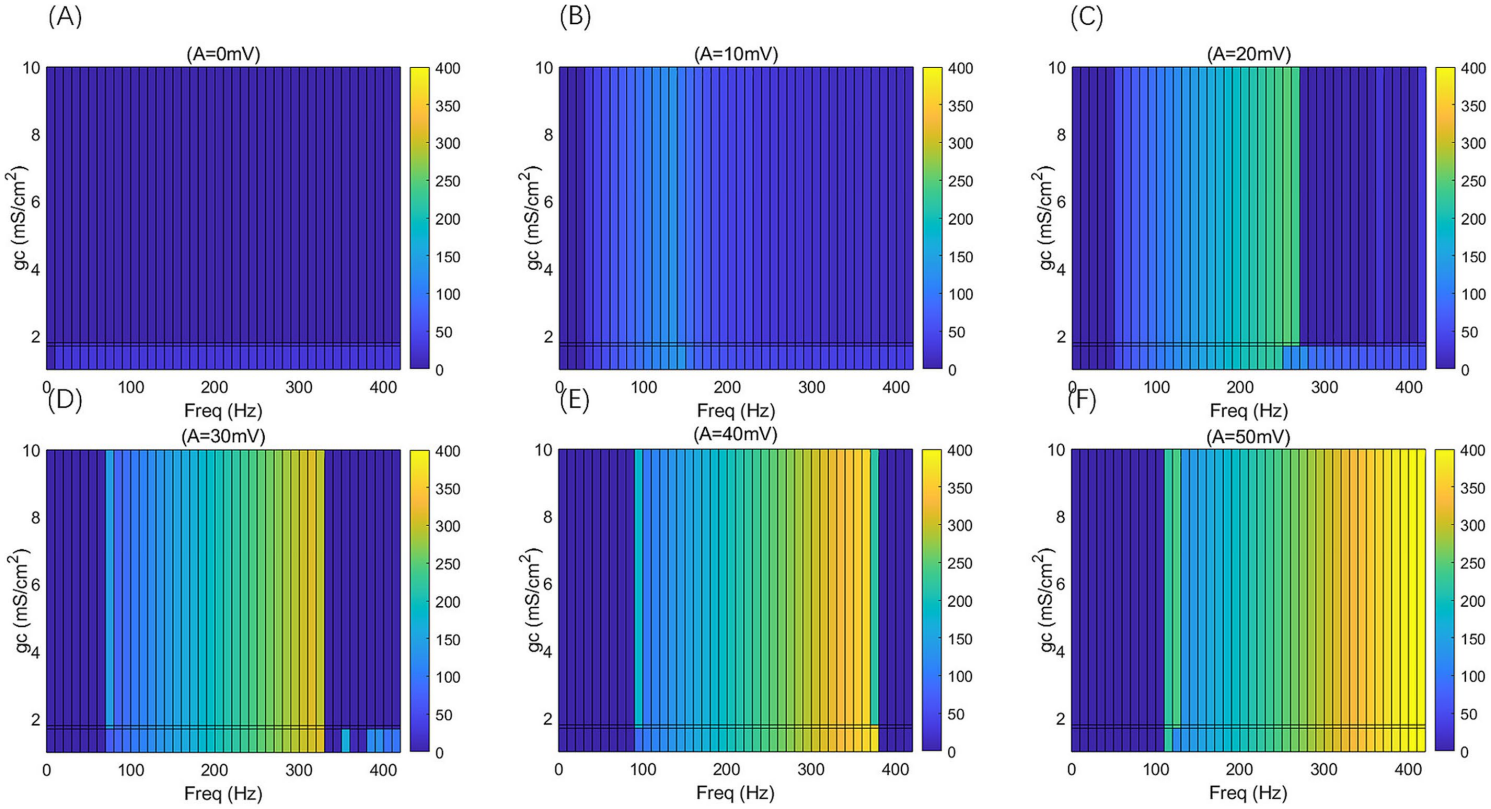
2D *F-g*_c_*-F*_req_ mapping of PR neurons under sinusoidal alternating electric field with different coupling conductance *g*_c_ and varying electric field amplitudes A, where *g*_c_ = 1, 1.7, 1.8, and 10 mS/cm^2^, and the electric field amplitudes *A* take values of 0, 10, 20, 30, 40, and 50 mV, with the frequency of the AC-IEF ranging from 0 to 420 Hz, with a 10 Hz interval.

When no AC-IEF is applied, the firing frequency of the neurons at different *g*_c_ values, as shown in Figures 6, 7 where *A* = 0 mV, remains unchanged with the frequency of the AC-IEF.

**Figure 8:**
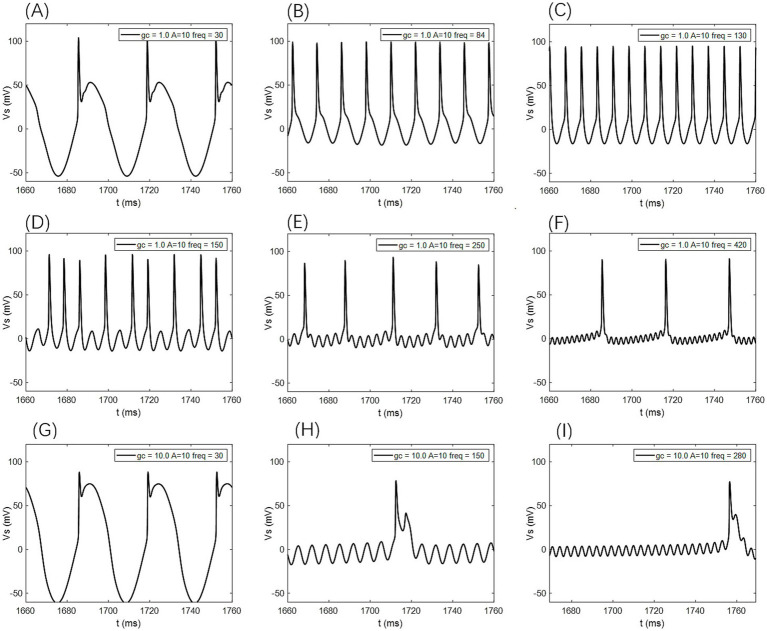
The firing states of PR neurons under AC-IEF with different coupling conductance *g*_c_ and varying field amplitudes are as follows: **(A)** periodic atypical burst firing state, **(B)** atypical spike firing state, **(C)** periodic spike firing state, **(D)** oscillatory-spike firing state, **(E)** periodic oscillatory-spike firing state, **(F)** periodic oscillatory-spike firing state, **(G)** periodic atypical burst firing state, **(H)** periodic oscillatory-burst firing state. **(I)** Periodic oscillatory-burst firing state.

When the amplitude of the AC-IEF is increased to 10 mV, the firing frequency of the neuron undergoes a significant change (as shown in Figures 6, 7 where *A* = 10 mV). Regardless of the *g*_c_ value, the neuron does not respond to the AC-IEF below 20 Hz. When the frequency of the applied AC-IEF lies within the range of 30–130 Hz, the neuronal firing frequency becomes entrained to the field frequency, exhibiting a 1:1 phase-locking relationship. As illustrated in Figure 6, this manifests as linear segments where the firing rate increases nearly proportionally with the stimulation frequency. Beyond this range, nonlinear dynamics dominate. The emergence of these locking effects can be attributed to the modulation of neuronal excitability by the external electric field, which induces entrainment of the firing rhythm to the field frequency (e.g., 1:1 or 2:1 phase locking). This entrainment mechanism provides a coherent explanation for the quasi-linear firing behavior observed within specific frequency ranges. In this range, the neuron gradually transitions from periodic atypical burst firing (Figure 8A) to periodic spike firing (from Figures 8B,C). As the electric field frequency continues to increase, the subthreshold oscillation phenomenon of the neuron increases with the rise in electric field frequency (as shown in Figures 8D-F). The firing frequency of the neuron also gradually decreases with the increase in electric field frequency, but the firing state remains in spike firing. Therefore, under these parameters, the frequency sensitivity range of the neuron to the AC-IEF is [30, 420] Hz. When the *g*_c_ value is 1.7 mS/cm^2^ or 1.8 mS/cm^2^, the neuron achieves a 1:1 phase-locking frequency range of [30, 130] Hz. In this range, the neuron gradually transitions from periodic atypical burst firing to periodic spike firing. After the electric field frequency exceeds the 1:1 phase-locking frequency, the firing state of the neuron is periodic oscillatory burst-spike alternating firing, and the firing frequency does not change after exceeding 260 Hz. Therefore, under these parameters, the sensitive frequency range is [20, 260] Hz. When the *g*_c_ value is 10 mS/cm^2^, the firing frequency of the neuron is 60 Hz at an electric field frequency of 30 Hz, achieving a 2:1 phase-locking, which is not observed for the other three values. Then, the neuron switches to 1:1 phase-locking within the electric field frequency range of [40, 140] Hz. The firing state at this time is shown in Figure 8G. When the electric field frequency is within the range of [110, 140] Hz, the firing state of the neuron is bi-periodic oscillatory burst firing (as shown in Figure 8H). As the electric field frequency continues to increase, the firing frequency of the neuron gradually converges, and the firing state gradually transitions to periodic atypical burst firing (as shown in Figure 8I). Therefore, under these parameters, the sensitive frequency range of the neuron is [30, 240] Hz.

When the amplitude of the applied induced electric field *A* increases to 20 mV (as shown in Figures 6, 7 for *A* = 20 mV), the neuron is not sensitive to AC-IEF frequencies below 40 Hz, regardless of the *g*_c_ value. When the *g*_c_ value is 1 mS/cm^2^, the electric field frequency range for achieving 1:1 phase-locking is [50, 210] Hz. Within this range, the neuron gradually transitions from periodic atypical burst firing to periodic spike firing. When the electric field frequency range is between [220, 240] Hz, although the firing frequency is not phase-locked, the neuron’s firing state remains periodic spike firing. As the induced electric field frequency continues to increase, the neuron enters a periodic oscillatory-spike firing state, and the firing frequency gradually decreases. The firing frequency changes little after the electric field frequency exceeds 370 Hz. Therefore, under these parameters, the sensitive frequency range of the neuron is [50, 370] Hz. When the *g*_c_ value is 1.7 mS/cm^2^ or 1.8 mS/cm^2^, the electric field frequency range for achieving 1:1 phase-locking is changed to [50, 230] Hz. Beyond the phase-locking frequency, the neuron transitions from periodic spike firing to periodic oscillatory-spike firing when the external field frequency is between [240, 260] Hz. After the frequency exceeds 260 Hz, for neurons with *g*_c_ equal to 1.8 mS/cm^2^, the firing state at an electric field frequency of 410 Hz (with *g*_c_ = 1.7 mS/cm^2^ at 390 Hz) is periodic oscillatory-burst-spike alternating firing. At other electric field frequencies, the neuron remains in an unstable state, exhibiting periodic oscillatory-spike firing immediately upon contact with the electric field, and after some time, transitioning to a subthreshold oscillation state, without returning to spike firing. When *g*_c_ is set to 10 mS/cm^2^, the neuron exhibits a 2:1 phase-locking pattern within the AC-IEF frequency range of [50, 70] Hz, maintaining a periodic atypical spike firing state. Subsequently, in the AC-IEF frequency range of [80, 190] Hz, the neuron transitions to a 1:1 phase-locking state, gradually shifting from periodic atypical burst firing to periodic spike firing. When the AC-IEF frequency is within [200, 280] Hz and at 310 Hz, the neuron remains in a subthreshold oscillatory state. When the AC-IEF frequency falls within [290, 300] Hz or exceeds 320 Hz, the neuron enters a periodic oscillatory-burst firing state. As the AC-IEF frequency further increases, the neuron’s firing frequency gradually converges. Therefore, under these conditions, the neuron’s sensitivity range spans [50, 190] Hz, [290, 300] Hz, and [320, 420] Hz.

When the applied electric field amplitude *A* is increased to 30 mV (as shown in Figures 6, 7 with *A* = 30 mV), the neuron does not respond to AC-IEF below 60 Hz, regardless of the *g*_c_ value. When *g*_c_ is set to 1 mS/cm^2^, the neuron achieves 1:1 phase-locking within the AC-IEF frequency range of [70, 300] Hz, gradually transitioning from periodic atypical burst firing to periodic spike firing. When the AC-IEF frequency falls within [310, 320] Hz, the neuron remains in a periodic spike firing state but no longer maintains 1:1 phase-locking. At AC-IEF frequencies of 330 Hz, 340 Hz, 360 Hz, and 370 Hz, the neuron shows a reduced firing frequency, initially entering a periodic oscillatory-spike firing state under AC-IEF stimulation. Over time, it transitions into a fully subthreshold oscillatory state and does not recover spike firing. Therefore, under these conditions, the neuron’s sensitivity range spans [70, 330] Hz ∪ [380, 420] Hz and includes 350 Hz. When *g*_c_ is set to 1.7 mS/cm^2^ or 1.8 mS/cm^2^, the neuron exhibits 2:1 phase-locking at an AC-IEF frequency of 70 Hz, characterized by periodic atypical burst firing. The neuron achieves 1:1 phase-locking within the AC-IEF frequency range of [80, 310] Hz, gradually transitioning from periodic atypical burst firing to periodic spike firing. When the AC-IEF frequency falls within [310, 320] Hz, the neuron remains in a periodic spike firing state but no longer maintains 1:1 phase-locking. When the AC-IEF frequency exceeds 330 Hz, the neuron fully transitions into a subthreshold oscillatory state. Therefore, under these conditions, the neuron’s sensitivity range to the AC-IEF spans [70, 330] Hz. When *g*_c_ is set to 10 mS/cm^2^, the neuron exhibits a 2:1 phase-locking pattern within the AC-IEF frequency range of [70, 110] Hz, gradually transitioning from periodic burst firing to periodic spike firing. Subsequently, in the AC-IEF frequency range of [120, 240] Hz, the neuron transitions to a 1:1 phase-locking state. When the AC-IEF frequency exceeds 250 Hz, the neuron’s firing state rapidly transitions into a subthreshold oscillatory state. Therefore, under these conditions, the neuron’s sensitivity range spans [70, 250] Hz.

When the applied electric field amplitude *A* is increased to 40 mV (as shown in Figures 6, 7 with *A* = 40 mV), the neuron does not respond to AC-IEF below 80 Hz, regardless of the *g*_c_ value. When *g*_c_ is set to 1 mS/cm^2^, the neuron exhibits 1:1 phase-locking within the AC-IEF frequency range of [90, 370] Hz, gradually transitioning from periodic burst firing to periodic spike firing. Once the AC-IEF frequency exceeds this phase-locking range, the neuron transitions into a subthreshold oscillatory state. Therefore, under these conditions, the neuron’s sensitivity range spans [80, 370] Hz. When *g*_c_ is set to 1.7 mS/cm^2^ or 1.81 mS/cm^2^, the neuron achieves 1:1 phase-locking within the AC-IEF frequency range of [100, 360] Hz, gradually transitioning from periodic atypical burst firing to periodic spike firing. At an AC-IEF frequency of 90 Hz, the neuron exhibits 2:1 phase-locking, characterized by periodic atypical burst firing. When the AC-IEF frequency falls within [360, 380] Hz, the neuron remains in a periodic spike firing state but no longer maintains 1:1 phase-locking. Once the AC-IEF frequency exceeds 380 Hz, the neuron fully transitions into a subthreshold oscillatory state. Therefore, under these conditions, the neuron’s sensitivity range to the AC-IEF spans [90, 380] Hz. When *g*_c_ is set to 10 mS/cm^2^, the neuron exhibits 2:1 phase-locking within the AC-IEF frequency range of [90, 120] Hz, maintaining a periodic atypical burst firing state. Subsequently, in the AC-IEF frequency range of [130, 280] Hz, the neuron transitions to a 1:1 phase-locking state, gradually shifting from periodic atypical burst firing to periodic spike firing. When the AC-IEF frequency exceeds 290 Hz, the neuron’s firing state rapidly transitions into a subthreshold oscillatory state. Therefore, under these conditions, the neuron’s sensitivity range spans [90, 290] Hz.

When the applied electric field amplitude *A* is increased to 50 mV (as shown in Figures 6, 7 with *A* = 50 mV), the neuron does not respond to AC-IEF below 100 Hz, regardless of the *g*_c_ value, and a 2:1 phase-locking phenomenon is observed. When *g*_c_ is set to 1 mS/cm^2^, the neuron exhibits 2:1 phase-locking at an AC-IEF frequency of 110 Hz, maintaining a periodic atypical burst firing state. The neuron achieves 1:1 phase-locking within the AC-IEF frequency range of [120, 410] Hz, during which it gradually transitions from periodic atypical burst firing to periodic spike firing. Beyond this phase-locking range, the neuron remains in a periodic spike firing state but no longer maintains 1:1 phase-locking. Therefore, under these conditions, the neuron’s sensitivity range spans [110, 420] Hz. When *g*_c_ is set to 1.7 mS/cm^2^ or 1.8 mS/cm^2^, the neuron exhibits 2:1 phase-locking within the AC-IEF frequency range of [110, 120] Hz, maintaining a periodic atypical burst firing state. For *g*_c_ = 1.7 mS/cm^2^, the neuron achieves 1:1 phase-locking within the AC-IEF frequency range of [130, 410] Hz, while for *g*_c_ = 1.8 mS/cm^2^, this range is [130, 400] Hz, with 410 Hz corresponding to a spike firing state that is not phase-locked to the AC-IEF. Within these ranges, the neuron gradually transitions from periodic atypical burst firing to periodic spike firing. Once the AC-IEF frequency exceeds 420 Hz, the neuron fully transitions into a subthreshold oscillatory state. Therefore, under these conditions, the neuron’s sensitivity range spans [120, 420] Hz. When *g*_c_ is set to 10 mS/cm^2^, the neuron exhibits 2:1 phase-locking within the AC-IEF frequency range of [110, 130] Hz, maintaining a periodic atypical burst firing state. Subsequently, in the AC-IEF frequency range of [140, 310] Hz, the neuron transitions to a 1:1 phase-locking state, gradually shifting from periodic atypical burst firing to periodic spike firing. When the AC-IEF frequency falls within [310, 330] Hz, the neuron remains in a periodic spike firing state but no longer maintains 1:1 phase-locking. Once the AC-IEF frequency exceeds 330 Hz, the neuron’s firing state rapidly transitions into a subthreshold oscillatory state. Therefore, under these conditions, the neuron’s sensitivity range spans [110, 330] Hz.

### 3.2 Effects of *V*_k_ on the firing sensitivity of neurons under a AC-IEF

**Figure 9:**
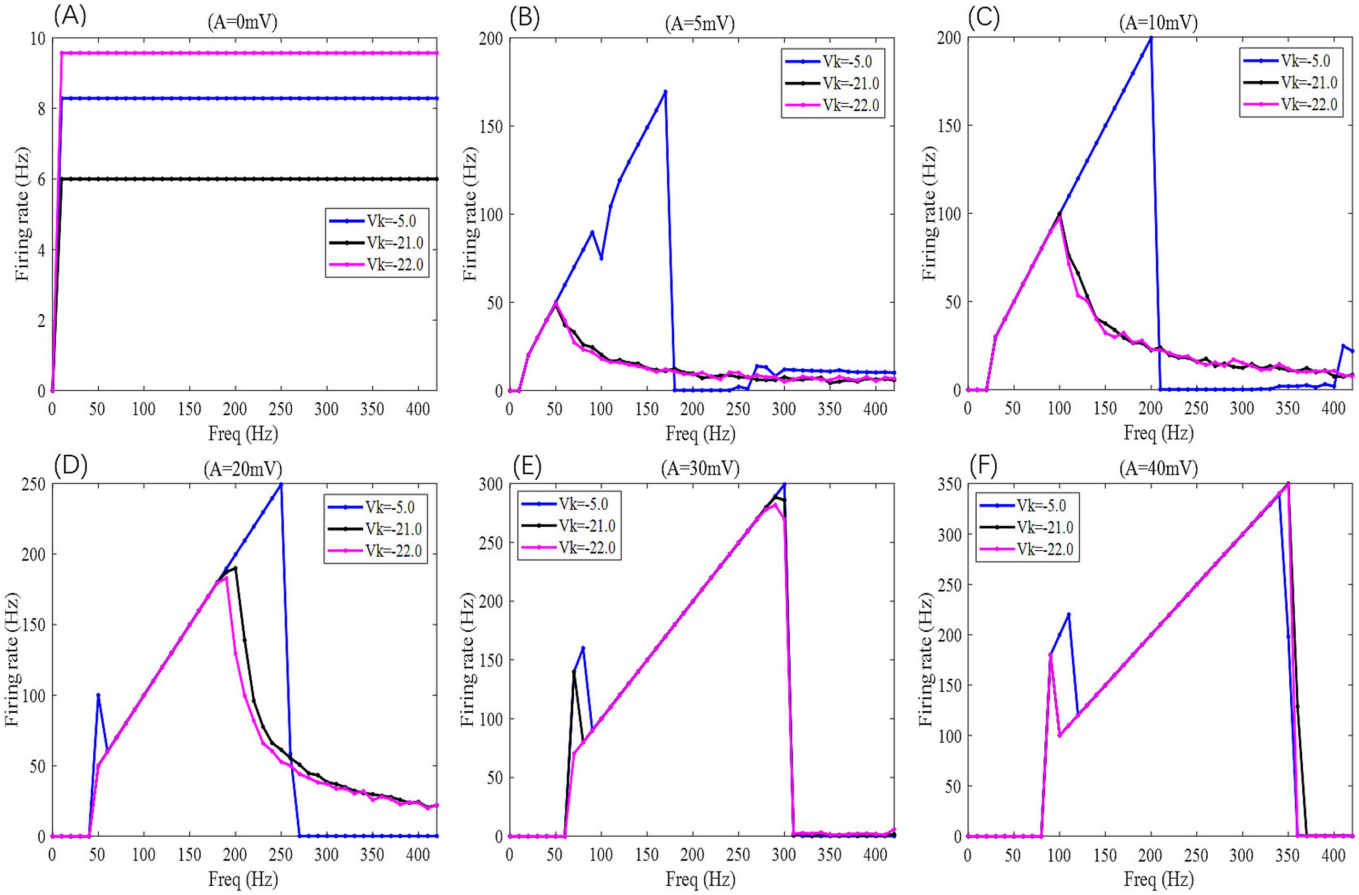
The *F-F*_req_ curves of the PR neuron under the influence of a sinusoidal AC-IEF at different potassium channel reversal potentials (*V*_k_ = −5, −21, −22 mV) and different electric field amplitudes (*A* = 0, 5, 10, 20, 30, 40 mV) are shown. The frequency of the AC-IEF ranges from 0 to 420 Hz with a 10 Hz interval.

**Figure 10:**
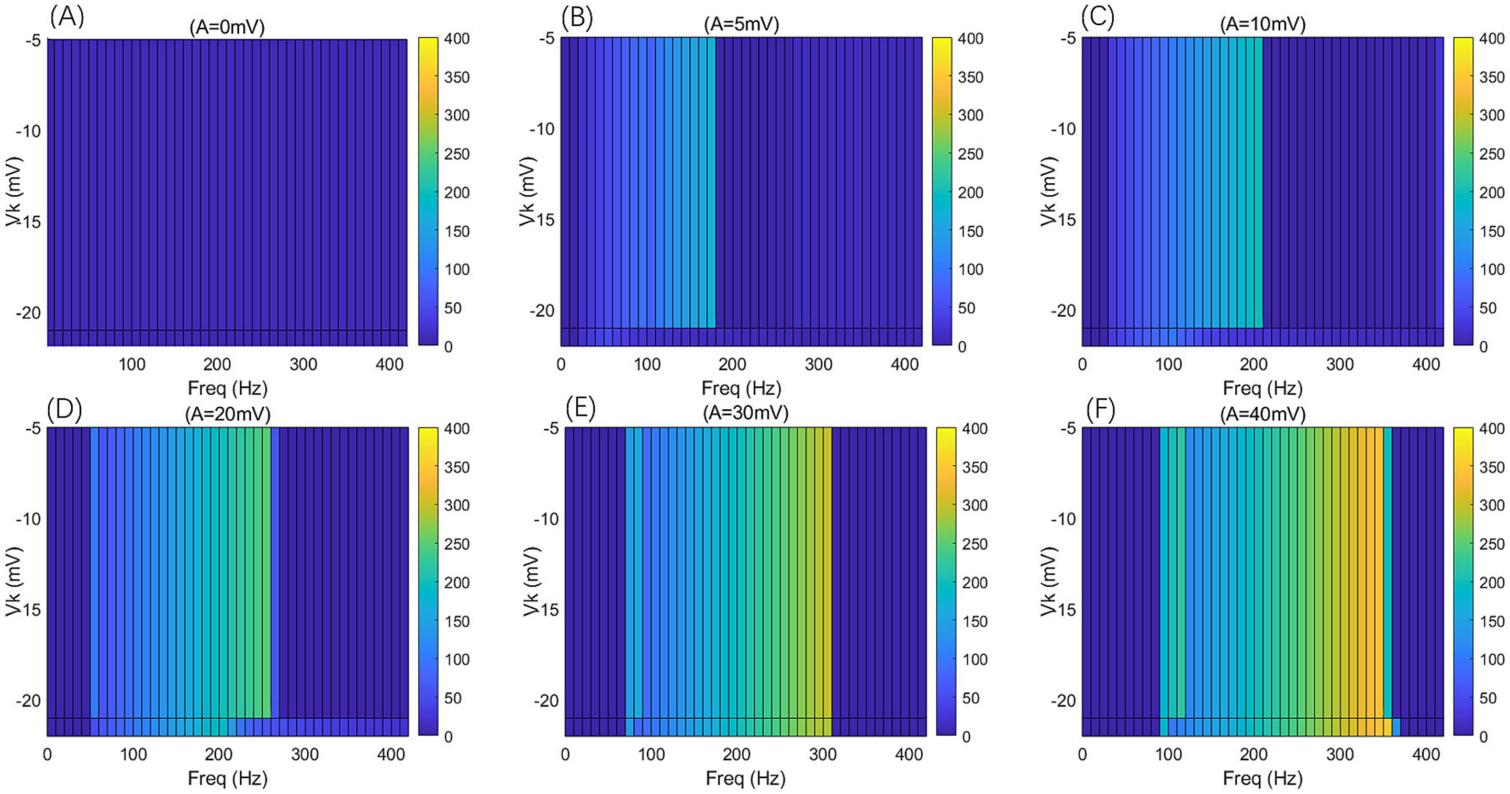
The *F-F*_req_ two-dimensional mapping of the PR neuron under the influence of a sinusoidal AC-IEF at different potassium channel reversal potentials (*V*_k_ = −5, −21, −22 mV) and different electric field amplitudes (*A* = 0, 5, 10, 20, 30, 40 mV) is presented. The AC-IEF frequency ranges from 0 to 420 Hz with a 10 Hz interval.

When no AC-IEF is applied, the firing frequency of the neuron at different bifurcation values of the potassium channel reversal potential *V*_k_ is shown in Figures 9, 10 with *A* = 0 mV. The firing frequency of the neuron remains unaffected by changes in the frequency of the AC-IEF.

**Figure 11:**
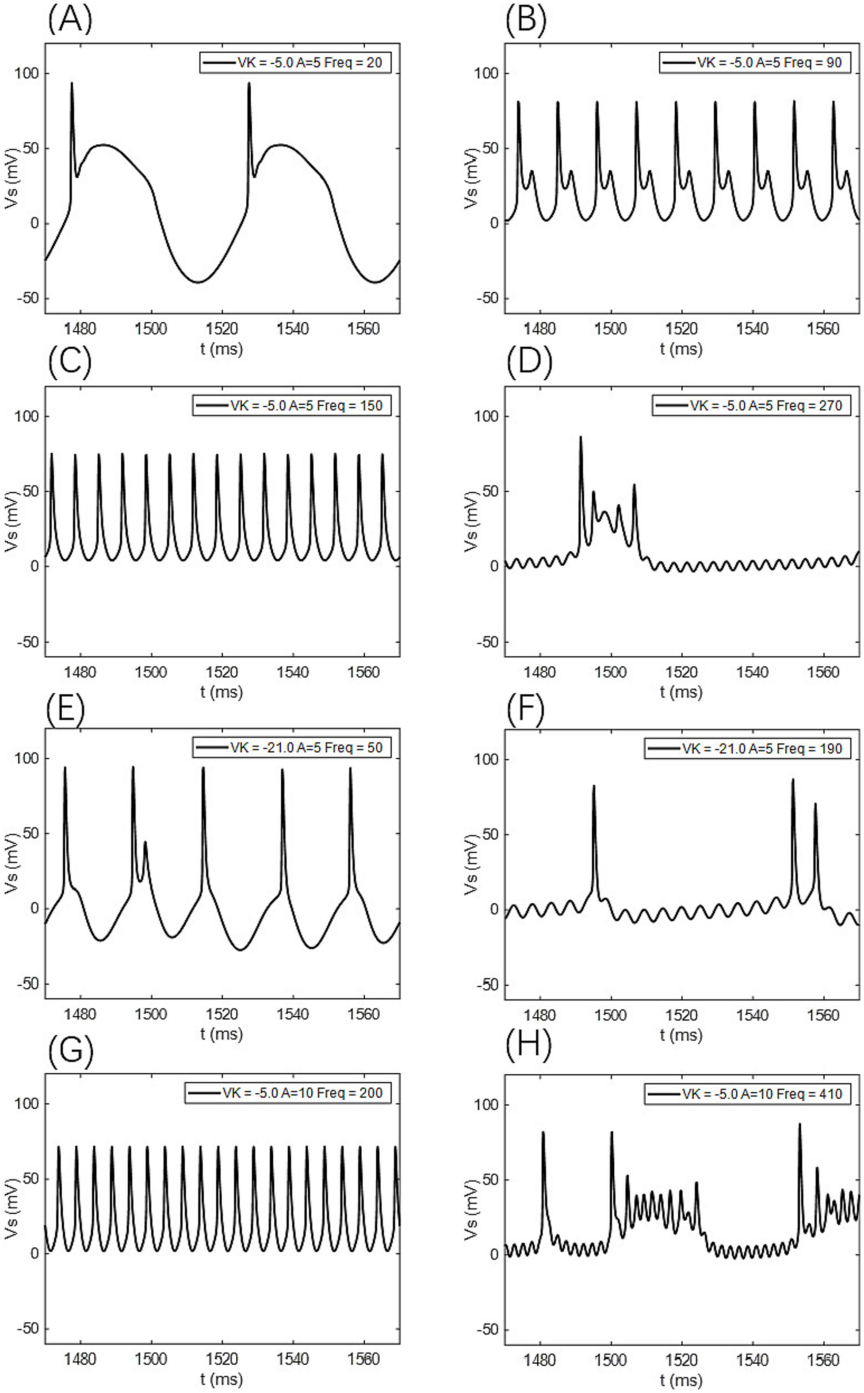
The firing states of the PR neuron under the influence of a sinusoidal AC-IEF at different potassium channel reversal potentials (*V*_k_) and electric field amplitudes (*A*). **(A)** Periodic atypical burst firing state, **(B)** atypical bi-periodic burst firing state, **(C)** periodic spike firing state, **(D)** oscillatory-burst firing state, **(E)** bi-periodic burst-spike alternating firing state, **(F)** oscillatory-bi-periodic burst-spike alternating firing state, **(G)** periodic spike firing state. **(H)** Oscillatory-burst-spike alternating firing state,

When the electric field amplitude *A* is increased to 5 mV, a significant change in the neuron’s firing frequency is observed (as shown in Figures 9, 10 with *A* = 5 mV). Regardless of the value of *V*_k_, the neuron does not respond to AC-IEF at frequencies below 10 Hz. When *V*_k_ is −5 mV, the neuron exhibits a 1:1 phase-locking between the electric field frequency and the neuron’s firing frequency in the frequency ranges of [20, 90] Hz and [120, 170] Hz. In the first range, the neuron transitions from a periodic atypical burst firing state (Figure 11A) to an atypical bi-periodic burst firing state (Figure 11B). In the second range, the neuron exhibits periodic spike firing (Figure 11C). When the electric field frequency ranges from [180, 260] Hz, the neuron enters a sub-threshold oscillatory state. Above 270 Hz, the neuron’s firing state shifts to an oscillatory-burst firing state (as shown in Figure 11D). The firing frequency gradually converges with increasing electric field frequency, and no further change is observed when the electric field frequency exceeds 350 Hz. Therefore, when the AC-IEF amplitude *A* is 5 mV and *V*_k_ is −5 mV, the neuron displays frequency sensitivity to the AC-IEF in the frequency intervals of [20, 180] Hz ∪ [270, 350] Hz. When *V*_k_ is −21 mV or −22 mV, the neuron achieves frequency locking within the electric field frequency range of [20, 50] Hz, where the firing state transitions from atypical burst firing to bi-periodic burst-spike alternating firing (Figure 11E). Once the electric field frequency exceeds the phase-locking frequency range, the firing frequency of the neuron gradually decreases, and the firing state transitions to an oscillatory-bi-periodic burst-spike alternating firing state (as shown in Figure 11F, with the burst firing phenomenon diminishing as the electric field frequency increases. After the electric field frequency exceeds 210 Hz, the firing frequency of the neuron remains almost constant. Therefore, with these parameters, the neuron’s frequency-sensitive range is [20, 210] Hz.

When the electric field amplitude *A* is increased to 10 mV (as shown in Figures 9, 10 with *A* = 10 mV), the neuron does not respond to AC-IEF below 20 Hz, regardless of the *V*_k_ value. When *V*_k_ is −5 mV, the neuron exhibits 1:1 phase-locking with the electric field frequency in the range of [30, 200] Hz. Within this range, the neuron transitions from periodic atypical burst firing to periodic spike firing (as shown in Figure 11G). When the electric field frequency ranges from [200, 400] Hz, the neuron is in a sub-threshold oscillatory state. Once the electric field frequency exceeds 410 Hz, the neuron enters an oscillatory-burst-spike alternating firing state (as shown in Figure 11H). Therefore, with these parameters, the neuron’s frequency-sensitive range is [30, 200] Hz ∪ [400, 420] Hz. When *V*_k_ is −21 mV or −22 mV, the neuron achieves frequency locking within the electric field frequency range of [30, 90] Hz. Within this range, the neuron transitions from periodic atypical burst firing to periodic spike firing. At a frequency of 100 Hz, the neuron remains in a spike firing state. Once the frequency exceeds 100 Hz, the neuron transitions to an oscillatory-burst-spike alternating firing state, with the firing frequency decreasing as the electric field frequency increases. Therefore, with these parameters, the neuron’s frequency-sensitive range is [30, 420] Hz.

When the electric field amplitude *A* is increased to 20 mV (as shown in Figures 9, 10 with *A* = 20 mV), the neuron does not respond to AC-IEF below 40 Hz, regardless of the *V*_k_ value. When *V*_k_ is −5 mV, the neuron achieves 2:1 phase-locking with its firing frequency at an electric field frequency of 50 Hz, and the firing state is periodic atypical burst firing. In the electric field frequency range of [60, 250] Hz, the neuron achieves 1:1 phase-locking with its firing frequency. Within this range, the firing state transitions from periodic atypical burst firing to periodic spike firing. At an electric field frequency of 260 Hz, the neuron remains in a periodic spike firing state but no longer exhibits 1:1 phase-locking. Once the electric field frequency exceeds 270 Hz, the neuron enters a sub-threshold oscillatory state. Therefore, with these parameters, the neuron’s frequency-sensitive range is [50, 270] Hz. When *V*_k_ is −21 mV or −22 mV, the neuron achieves frequency locking within the electric field frequency range of [50, 180] Hz. Within this range, the neuron transitions from periodic atypical burst firing to periodic spike firing. After this range, the neuron transitions from periodic spike firing to an oscillatory-burst-spike alternating firing state as the electric field frequency increases, with the firing frequency gradually decreasing. Therefore, with these parameters, the neuron’s frequency-sensitive range is [50, 420] Hz.

When the electric field amplitude *A* is increased to 30 mV (as shown in Figures 9, 10 with *A* = 30 mV), the neuron does not respond to AC-IEF below 60 Hz, regardless of the *V*_k_ value. When *V*_k_ is −5 mV, the neuron achieves 2:1 phase-locking with its firing frequency in the electric field frequency range of [70, 80] Hz, with the firing state being periodic atypical burst firing. In the electric field frequency range of [90, 300] Hz, the neuron achieves 1:1 phase-locking with its firing frequency. Within this range, the firing state transitions from periodic atypical burst firing to periodic spike firing. Once the electric field frequency exceeds 310 Hz, the neuron enters a sub-threshold oscillatory state. Therefore, with these parameters, the neuron’s frequency-sensitive range is [70, 310] Hz. When *V*_k_ is −21 mV, the neuron achieves 2:1 phase-locking with its firing frequency at an electric field frequency of 70 Hz, with the firing state being periodic atypical burst firing. In the electric field frequency range of [80, 290] Hz, the neuron achieves 1:1 phase-locking with its firing frequency. Within this range, the firing state transitions from periodic atypical burst firing to periodic spike firing. At an electric field frequency of 300 Hz, the neuron remains in periodic spike firing but no longer exhibits 1:1 phase-locking. Once the electric field frequency exceeds 310 Hz, the neuron enters a sub-threshold oscillatory state. Therefore, with these parameters, the neuron’s frequency-sensitive range is [70, 310] Hz. When *V*_k_ is −22 mV, no 2:1 phase-locking occurs. In the electric field frequency range of [70, 270] Hz, the neuron achieves 1:1 phase-locking with its firing frequency. Within this range, the firing state transitions from periodic atypical burst firing to periodic spike firing. At an electric field frequency of [280, 300] Hz, the neuron remains in periodic spike firing but no longer exhibits 1:1 phase-locking. Once the electric field frequency exceeds 310 Hz, the neuron enters a sub-threshold oscillatory state.

When the electric field amplitude *A* is increased to 40 mV (as shown in Figures 9, 10 with *A* = 40 mV), the neuron does not respond to AC-IEF below 80 Hz, regardless of the *V*_k_ value. When *V*_k_ is −5 mV, the neuron achieves 2:1 phase-locking with its firing frequency in the electric field frequency range of [90, 110] Hz, with the firing state being periodic atypical burst firing. In the electric field frequency range of [120, 340] Hz, the neuron achieves 1:1 phase-locking with its firing frequency. Within this range, the firing state transitions from periodic atypical burst firing to periodic spike firing. At an electric field frequency of 350 Hz, the neuron remains in periodic spike firing but no longer exhibits 1:1 phase-locking. Once the electric field frequency exceeds 360 Hz, the neuron enters a sub-threshold oscillatory state. Therefore, with these parameters, the neuron’s frequency-sensitive range is [90, 360] Hz. When *V*_k_ is −21 mV or −22 mV, the neuron achieves 2:1 phase-locking with its firing frequency at an electric field frequency of 90 Hz, with the firing state being periodic atypical burst firing. In the electric field frequency range of [100, 350] Hz, the neuron achieves 1:1 phase-locking with its firing frequency. Within this range, the firing state transitions from periodic atypical burst firing to periodic spike firing. Once the electric field frequency exceeds the neuron’s firing cutoff frequency (370 Hz for *V*_k_ = −21 mV and 360 Hz for *V*_k_ = −22 mV), the neuron enters a sub-threshold oscillatory state. When *V*_k_ is −21 mV, the neuron remains in periodic spike firing at an electric field frequency of 370 Hz. Therefore, when *V*_k_ is −21 mV, the neuron’s frequency-sensitive range is [90, 370] Hz, and when *V*_k_ is −22 mV, the neuron’s frequency-sensitive range is [90, 360] Hz.

When the electric field amplitude *A* is increased to 50 mV, the firing trend remains consistent with that at *A* = 40 mV, with the only difference being the different intervals of firing states. As a result, no further detailed analysis will be provided.

### 3.3 Pattern analysis of *g*_c_ and *V*_k_ influences on neuronal firing under AC-IEF

An improved PR neuron model incorporating AC-IEF was established based on the standard PR two-compartment neuron model. The study explores the values of conductance (*g*_c_) and potassium channel reversal potential (*V*_k_) when the neuron is in a bifurcation state under weak DC stimulation. Based on these values, the effect of external AC-IEF amplitude and frequency on the firing state and frequency of the neuron in the bifurcation state was investigated, and the sensitive intervals of AC-IEF frequency for different bifurcation values under various field amplitudes were analyzed. The conclusions derived from the simulation analysis are as follows:

① Regardless of the values of *g*_c_ or *V*_k_, the neuron does not show any firing state or firing sensitivity to the AC-IEF frequency (in Hz) below twice the amplitude of the AC-IEF (in mV).
② Neurons exhibit sensitivity to the amplitude of external AC-IEF; as the amplitude of the external AC-IEF increases, the maximum firing frequency of the neurons under the AC-IEF also increases, leading to a broader sensitive range. However, the forms of firing states exhibited by neurons at different frequencies are limited.
③ The inclusion of the AC-IEF causes the neuron to exhibit 1:1 or 2:1 phase locking between the firing frequency and the electric field frequency. Furthermore, as the amplitude of the AC-IEF increases, the frequency range over which phase locking occurs gradually widens.
④ When the amplitude of the AC-IEF is below 30 mV, the firing pattern of the neuron under the electric field changes with the electric field frequency in accordance with the value of *g*_c_. When *g*_c_ is 1 mS/cm^2^, the firing pattern of the neuron begins with a resting state, transitions to periodic atypical burst firing, then changes to periodic atypical spike firing, followed by periodic spike firing, and eventually becomes oscillatory-spike firing. When *g*_c_ is 1.7 mS/cm^2^ or 1.8 mS/cm^2^, the firing pattern begins with a resting state, progresses to periodic atypical burst firing, then periodic atypical spike firing, followed by periodic spike firing, and finally transitions to oscillatory-burst-spike alternating firing. When *g*_c_ is 10 mS/cm^2^, the firing pattern starts with a resting state, progresses through periodic atypical burst firing, periodic atypical spike firing, and periodic spike firing, ultimately evolving into oscillatory-burst-spike alternating firing.
⑤ When the amplitude of the AC-IEF is greater than or equal to 30 mV, the firing patterns of the neuron under the electric field are the same regardless of the *g*_c_ value. The firing pattern follows this sequence: it starts from a resting state, transitions to periodic atypical burst firing, followed by periodic atypical spike firing, changes to periodic spike firing, and finally enters sub-threshold oscillation.
⑥ When the *V*_k_ value is −5 mV, the firing frequency of the neuron with an electric field amplitude greater than or equal to 15 mV varies with the electric field frequency. Initially, the neuron is in a resting state, followed by periodic atypical burst firing, then periodic atypical spike firing, transitioning into periodic spike firing, and finally into a subthreshold oscillatory state. When the electric field amplitudes are 5 mV and 10 mV, the firing states of the neuron eventually become periodic oscillatory-burst firing and oscillatory-burst-spike alternating firing, respectively, while the other firing patterns remain consistent with those when the electric field amplitude is greater than 15 mV.
⑦ When the *V*_k_ value is −21 mV or −22 mV, the firing frequency of the neuron with an electric field amplitude less than 30 mV changes with the electric field frequency as follows: the neuron starts in a resting state, followed by periodic atypical burst firing, then periodic atypical spike firing, transitioning into periodic spike firing, and finally into oscillatory-burst-spike alternating firing. When the electric field amplitude is greater than or equal to 30 mV, the firing frequency of the neuron changes with the electric field frequency as follows: initially in a resting state, followed by periodic atypical burst firing, then periodic spike firing, and finally transitioning into a subthreshold oscillatory state.

**Figure 12:**
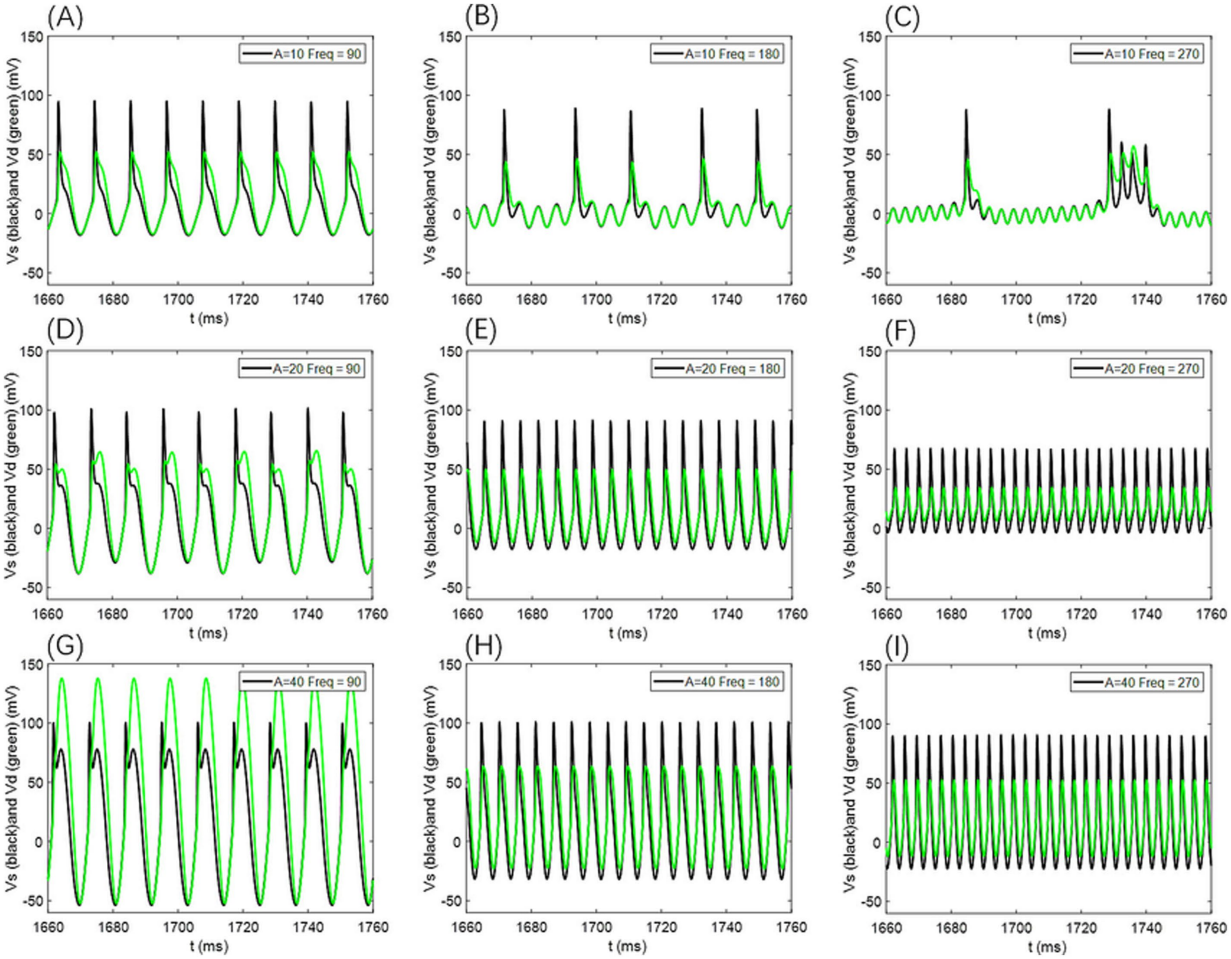
Comparison of neuronal cell membrane potential (*V*_s_) and dendritic membrane potential (*V*_d_) under different AC-induced electric field amplitudes *A* and electric field frequencies *F*_req_. Model parameters are *ρ* = 0.5, *g*_c_ = 2.1, and *V*_k_ = −15.

In our simulations, *V*_s_ and *V*_d_ exhibit no significant phase differences (as shown in Figure 12); their firing states and temporal dynamics remain consistent, with only amplitude and waveform variations. This suggests strong coupling between the cell body and dendrites under the current parameters, although phase lags may occur in models with weaker coupling or extended dendritic structures.

**Figure 13:**
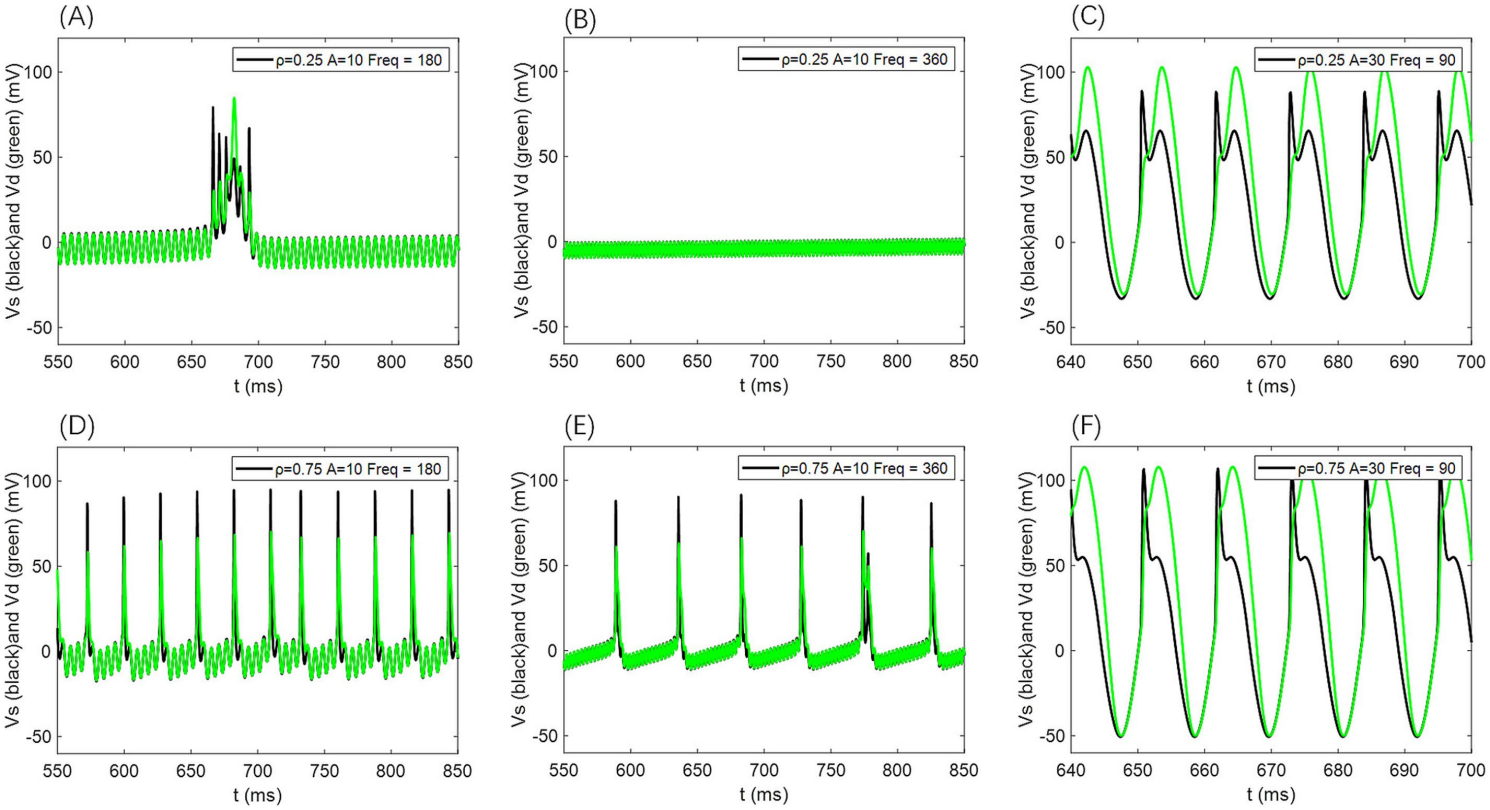
Comparison of neuronal cell membrane potential (*V*_s_) and dendritic membrane potential (*V*_d_) under different AC induced electric field amplitudes *A* and field frequencies *F*_req_. Model parameters are *ρ* = 0.25 or 0.75, *g*_c_ = 2.1, and *V*_k_ = −15.

For additional simulations at *ρ* = 0.25 and *ρ* = 0.75, the voltage traces from the somatic and dendritic compartments likewise showed no appreciable phase differences (as illustrated in Figure 13); only amplitude variations were evident, further supporting that the absence of phase lag remains robust across different *p*-values.

## 4 Discussion

### 4.1 Limitations

The present study advances our understanding of how alternating current inhomogeneous electric fields (AC-IEF) modulate neuronal excitability, with results that align with and extend prior research. Consistent with Yan et al. (2020) and Asan et al. (2020), we observed that AC-IEF can effectively alter neuronal firing rates through modulation of membrane potential and ion channel kinetics, and that its effects are highly sensitive to structural coupling between somatic and dendritic compartments. Similar to the findings of Bertagna et al. (2021), our analysis highlights the crucial role of calcium channel dynamics in shaping field responsiveness. Furthermore, in line with Kafraj et al. (2020), our simulations reveal nonlinear behaviors such as frequency-dependent modulation and firing pattern transitions, reinforcing the notion that neurons adaptively reorganize their activity in response to subtle external perturbations. While earlier studies have focused on cortical (Handler and Ginty, 2021) or mechanosensory neurons, our work leverages the two-compartment Pinsky–Rinzel (PR) model to characterize somato-dendritic coupling under heterogeneous AC-IEF stimulation, thereby providing a fine-grained view of subregional response dynamics. This modeling framework complements the multi-scale approaches described by Mokhtare et al. (2022) and Alfihed et al. (2024), linking cellular mechanisms to system-level modulation strategies.

Despite these contributions, several limitations must be acknowledged. First, our analysis is based on a single biophysically grounded PR neuron model, which may not capture the full diversity of responses across neuron types or morphologies. Second, the AC-IEF was simplified as a sinusoidal voltage input without accounting for spatial gradients or tissue-level inhomogeneities present *in vivo*. Third, we focused on firing rate and bifurcation dynamics, leaving other important aspects—such as spike timing, phase locking, and network-level interactions—unexplored. Lastly, our parameter sensitivity analysis was limited to gcg_cgc and VkV_kVk, without examining possible co-regulatory effects from other intrinsic or synaptic conductances. Addressing these limitations in future studies, particularly by incorporating multi-compartment and network-scale models, will be essential for developing more comprehensive and physiologically realistic descriptions of neuronal sensitivity to AC-IEF.

### 4.2 Implications

This work provides a quantitative framework to understand how neurons respond to sinusoidal AC-IEF in a frequency- and amplitude-dependent manner. The identification of firing rate sensitivity patterns and bifurcation behaviors under different field parameters enriches our understanding of resonance-like phenomena in single-neuron dynamics, which have been increasingly recognized in both modeling and electrophysiological studies (Schmidt et al., 2014; Toloza et al., 2018). By systematically mapping the firing rate against intrinsic parameters (*g*_c_, *V*_k_) and stimulation characteristics (amplitude and frequency), this study reveals regimes where external fields can selectively enhance, suppress, or rhythmically entrain neuronal firing—mechanisms that have been linked to both physiological oscillations and therapeutic modulation (Boyle and Fröhlich, 2013; Negahbani et al., 2018).

In addition, since oscillatory inputs were employed to drive the neuronal membrane, it is relevant to consider the potential role of stochastic resonance (SR) and signal-to-noise ratio (SNR) in shaping the observed dynamics. Stochastic resonance refers to the counterintuitive phenomenon in which the presence of moderate noise can enhance the detection or transmission of weak oscillatory signals in nonlinear systems, including neurons. In this context, variations in firing rate sensitivity across different stimulation regimes may reflect noise-assisted amplification of subthreshold oscillatory inputs, thereby increasing the effective SNR of neuronal responses. Such mechanisms have been suggested to underlie improved information transfer and resonance-like behaviors in excitable membranes, further linking the present findings to broader theories of neural coding and oscillatory signal processing.

These findings provide mechanistic insight into the effects of weak electric fields, thereby supporting ongoing efforts to optimize non-invasive brain stimulation techniques such as transcranial alternating current stimulation (tACS). Recent studies have demonstrated that tACS can modulate cortical excitability and network activity by interacting with the brain’s intrinsic oscillatory properties (Liu et al., 2018; Krause et al., 2019).

### 4.3 Future directions

Future research should extend this modeling framework to multicompartmental or morphologically detailed neurons to evaluate the spatial sensitivity of dendritic and somatic compartments to AC-IEF. Incorporating heterogeneous populations or networks of neurons would allow exploration of synchronization, population-level resonance, and emergent behaviors under electric field stimulation. Moreover, integrating more realistic field models—such as those derived from finite-element simulations of head tissues—could bridge the gap between theoretical predictions and clinical applications. Finally, examining the interplay between AC-IEF and synaptic inputs, neuromodulators, or pathological perturbations (e.g., ion channel dysfunction) could reveal new avenues for targeted neuromodulation in neurological disorders.

## 5 Conclusion

This study explores the firing activity and sensitivity of two-compartment neurons under the influence of an AC-IEF. Using systematic modeling and numerical simulations, this study examines how different neuronal parameters influence the complex relationships between firing patterns and firing frequencies under varying electric field strengths and frequencies. These findings provide new insights into the dynamic behavior of neurons under external electric fields and lay the foundation for future neuromodulation technologies. This study demonstrates that AC-IEF can significantly affect neuronal firing activity, highlighting their potential for developing electric field-based neuromodulation approaches. Furthermore, it is important to note that the external drive used in this study was implemented using a half-wave rectified sine wave, whose amplitude is inversely proportional to frequency. Therefore, the amplitude-frequency thresholds reported should be interpreted qualitatively rather than quantitatively predicted.

## Data Availability

The original contributions presented in the study are included in the article/supplementary material, further inquiries can be directed to the corresponding authors.
